# Microbes and Climate Change: a Research Prospectus for the Future

**DOI:** 10.1128/mbio.00800-22

**Published:** 2022-04-19

**Authors:** James M. Tiedje, Mary Ann Bruns, Arturo Casadevall, Craig S. Criddle, Emiley Eloe-Fadrosh, David M. Karl, Nguyen K. Nguyen, Jizhong Zhou

**Affiliations:** a Center for Microbial Ecology, Michigan State University, East Lansing, Michigan, USA; b Department of Ecosystem Science and Management, The Pennsylvania State University, University Park, Pennsylvania, USA; c Department of Molecular Microbiology and Immunology, Johns Hopkins Bloomberg School of Public Health, Baltimore, Maryland, USA; d Department of Civil and Environmental Engineering, Stanford University, Stanford, California, USA; e DOE Joint Genome Institute, Lawrence Berkeley National Laboratory, Berkeley, California, USA; f Daniel K. Inouye Center for Microbial Oceanography: Research and Education, University of Hawai‘i at Mānoa, Honolulu, Hawaii, USA; g American Academy of Microbiology, American Society for Microbiology, Washington, DC, USA; h Institute for Environmental Genomics, University of Oklahoma, Norman, Oklahoma, USA; Georgia Institute of Technology School of Biological Sciences

## Abstract

Climate change is the most serious challenge facing humanity. Microbes produce and consume three major greenhouse gases—carbon dioxide, methane, and nitrous oxide—and some microbes cause human, animal, and plant diseases that can be exacerbated by climate change. Hence, microbial research is needed to help ameliorate the warming trajectory and cascading effects resulting from heat, drought, and severe storms. We present a brief summary of what is known about microbial responses to climate change in three major ecosystems: terrestrial, ocean, and urban. We also offer suggestions for new research directions to reduce microbial greenhouse gases and mitigate the pathogenic impacts of microbes. These include performing more controlled studies on the climate impact on microbial processes, system interdependencies, and responses to human interventions, using microbes and their carbon and nitrogen transformations for useful stable products, improving microbial process data for climate models, and taking the One Health approach to study microbes and climate change.

## OPINION/HYPOTHESIS

Climate change is now widely recognized as the most serious contemporary challenge for humanity. Indeed, a new report from the Intergovernmental Panel on Climate Change (IPCC) states that the situation has grown even worse, with 3.3 billion of the world’s population highly vulnerable to climate change, and that current unsustainable development patterns are increasing exposure of ecosystems and people to climate hazards (1). We can engage in solutions to change from the current trajectory as individuals, as action leaders for society, and as microbiologists with domain expertise. Microbes have prominent roles related to climate change. They produce and consume the three dominant gases that are responsible for 98% of the increased warming: carbon dioxide (CO_2_), methane (CH_4_), and nitrous oxide (N_2_O). While microbes are sources of these gases as part of natural processes, some of their recent increase is due to changes in human activities that result in microbes having more access to carbon and nitrogen that they convert into these three products. Our actions can be to understand and then implement practices that mitigate microbial activities to decelerate the production of these gases, such as reduced soil tillage, or use microbes to repurpose waste carbon or nitrogen into useful and stable products.

Fortunately, microbes also consume these three gases and do so when their growth conditions favor the use of these gases as resources, namely, photo- or chemoautotrophic growth (cyanobacteria, algae, nitrifiers), methanotrophy (methane oxidizers), and nitrous oxide reduction (denitrifiers). The environmental conditions and interactions of these microbes, often influenced by humans, determine whether they carry out production or consumption of these gases. In some cases, we can manage conditions to favor microbial consumption of these gases.

Microbes that produce and consume these gases live in so many different habitats, and these habitats have very different spatial scales and process times, making it a challenge to quantify their contributions and changes in response to environmental conditions, e.g., warming, storms, and drought. Three ecosystem types pose three distinct assessment and management challenges: terrestrial, ocean, and urban. Improved measurements and models are keys to determine greenhouse gas (GHG) fluxes in these different systems, at different scales, and their patterns of change in response to human actions.

Beyond microbes’ direct role with GHG, other microbes with pathogenic potential respond to climate change by having their ranges extended via insect vectors, flooding, or severe storms, and hosts affected by heat or drought may become more vulnerable, whether they be human, animal, or plant. Among the complex climate change phenomena are cascading effects that can be difficult to manage or even predict. For example, a severe storm from more extreme weather can cause sewage overflow that disperses and mixes pathogens and problematic antibiotic resistances into waterways, which can greatly expand the pathogenic microbes’ range and their chance for horizontal gene exchange. This can result in multidrug-resistant pathogens reaching drinking water, food crop irrigation, or swimming beaches.

The American Academy of Microbiology hosted a colloquium on 5 November 2021 to discuss the evolving relationship between climate change, microbes, and the cascading effects. The authors participated in the colloquium. This paper builds on some concepts discussed at the colloquium and provides an extended view and opinions on some of the needed research to fill the knowledge gaps.

## INTERDEPENDENT DYNAMIC OF CLIMATE CHANGE AND MICROBES IN DIFFERENT ECOSYSTEMS

### Terrestrial environments.

Although soil microbes play vital roles in regulating Earth’s climate by controlling the turnover of soil organic matter (SOM), the largest organic carbon pool in the terrestrial biosphere, our understanding of how climate change affects soil microbes and how they regulate Earth’s climate is very limited (2). Various studies based on topsoil demonstrate that climate warming leads to divergent succession of grassland microbial communities, accelerates microbial temporal scaling, reduces microbial diversity, increases network complexity and stability, stimulates soil respiration and SOM decomposition, lowers respiratory temperature sensitivity, and shows no effects on soil carbon storage (3–9). Despite these core discoveries, it is not clear whether such experimental observations are broadly applicable to other terrestrial biomes and over longer ecological time.

From both theoretical and empirical perspectives, it is expected that the impacts of climate change on soil microbes would vary substantially across different ecosystems, primarily due to the huge spatial heterogeneity of terrestrial ecosystems in climate, plant diversity and composition, soil physics, chemistry, soil microbial community composition and structure, and evolutionary history. We also hypothesize that the effects of climate change on soil microbes will not show a linear increase or decrease over longer ecological times because ecosystems under natural settings are complex, their responses are nonlinear, and their dynamics are time dependent.

To search for general patterns of the feedback responses of microbes to climate change, microbe-centric, multifactor climate change experiments under realistic field settings are urgently needed across various ecosystems on different continents. This is because manipulated experiments are the most effective way to separate the effects of climate change factors from confounding environmental variations and allow us to quantify the responses and feedbacks of terrestrial ecosystems to anthropogenic perturbations (10). Although many manipulated climate change experiments in different ecosystems are available, they have been conducted at single sites and thus represent responses under only one set of site conditions. Also, nonsystematic soil sampling has been carried out in the majority of these sites because such experiments are often established as plant centric, which restricts destructive soil sampling. Consequently, information on the temporal dynamics of microbes in response to climate change is rarely available. Such microbe-centric experiments would allow us to examine the responses of different soil microbes (e.g., bacteria, archaea, fungi, protists, and viruses) and microfauna in both topsoil and subsoil to multiple climate change factors (e.g., warming, elevated CO_2_, drought, increased precipitation, nutrient addition, and their interactions). Such experiments would also allow us to collect systematic time-series soil samples (e.g., initially, weekly, monthly, seasonally, and yearly) and associated ecosystem process data (e.g., plant productivity, soil respiration, soil carbon dynamics, and nutrient status). These data are essential for advanced mathematical tools (e.g., generalized Lotka-Volterra modeling, empirical dynamic modeling, and deep learning) to predict their nonlinear dynamics and disentangle the underlying community assembly mechanisms, particularly the biotic interactions among different microbes, kingdoms (e.g., plants, soil fauna, and microbes), and their importance to ecosystem functioning.

To reduce experimental cost, such microbe-centric experiments should be leveraged with existing infrastructures such as university research stations, the National Ecological Observatory Network (NEON), and Long-Term Ecological Research (LTER), the last two of which are U.S.-based, continental-scale, complementary ecological research sites (11). Also, a global consortium should be established to coordinate research efforts by having identical/consistent experimental treatments, sampling protocols, and measurements (11, 12).

Finally, with reliable long-term systematic data on microbial dynamics and relevant ecosystem functional processes from representative ecosystems and environments worldwide, consensus patterns and possible general rules on the feedback responses of microbes to climate change can be obtained. Such information can be incorporated into terrestrial ecosystem models and/or Earth system models (ESMs) to scale our understanding from individual sites to regional, continental, and global (6, 13).

### Ocean environments.

The global ocean covers ~70% of the planet to an average depth of 4,000 m. Oceanic habitats are physically and chemically diverse, with greater than 50 biomes from the tropics to the poles and from the sunlit surface layer to the dark abyss (14). Each biome supports a unique microbe-based ecosystem that forms a complex adaptive system (15), with emergent processes and services that are inextricably linked to habitat variability. With nearly 4 billion years of evolutionary history, oceanic microbes have adapted to a constantly changing planet and have developed physiological plasticity and resiliency that may confer some protection against human-induced climate change. However, the current rates of climate change resulting from heat-trapping greenhouses gases are higher than at any other time in Earth’s history and, therefore, represent a great threat to the microbial inhabitants of the sea.

Oceans play a critical role in global climate dynamics. They absorb >90% of the heat accumulating in the atmosphere and have absorbed ~25% of the excess carbon dioxide since the industrial revolution, the latter leading to ocean acidification. A warmer, more stratified ocean also leads to deoxygenation, and all three threats are consequences of excess carbon dioxide emissions from human activities (16). In addition, enhanced stratification will accelerate the pace of future warming. In the past century, and especially in the past 2 decades, marine heat waves have been observed with increasing frequency and duration in all major ocean basins (17, 18), and they are expected to increase due to anthropogenic climate change. These prolonged periods (months) of anomalously high sea surface temperature over large regions (thousands of kilometers) have led to mass mortalities of marine life, including photosynthetic microorganisms. Rapid habitat changes, like those resulting from marine heat waves, may threaten global biodiversity and force oceanic habitats into alternate, less desirable ecosystem states with a lower overall resiliency for future change.

Because the ecological impacts of ocean warming and acidification are essentially irreversible on time scales of centuries (19), there is a scientific imperative to develop a comprehensive understanding of microbes and climate change. Due to its global expanse, most of the ocean is relatively inaccessible, so direct measurements of climate change impacts on microbial processes are limited to only a few long-term ocean observatories (20) that will facilitate a more comprehensive understanding of climate change microbial oceanography. These open-ocean sentinels provide the observational data sets that are required for the unambiguous detection of climate change impacts and for the development of Earth system models to predict the state of future oceans. New biogeochemical models will need to consider the resiliency of diverse microbial communities, as well as the interactions of multiple drivers, to accurately predict the impacts of climate change. In this regard, observations and perturbation experiments using natural microbial communities are essential since unispecies laboratory studies will never capture the adaptation and evolutionary potentials of sea microbes (21). Detailed studies of the microbial ecology of marine heat waves would be an excellent component of this future research prospectus.

Finally, the relatively new discipline of intervention ecology is based on the premise that humans may be able to alter the direction of climate change to facilitate the restoration of natural ecosystems currently under threat (22). A recent National Academies report on the feasibility, cost, and potential impacts of ocean-based carbon dioxide removal provides a basic research blueprint for the restoration of marine ecosystems and the services they provide (23).

### Urban environments.

The rise of megacities with large carbon footprints is a driving force for feedback loops that exacerbate the negative impacts of climate change, such as disease outbreaks. An attractive economic response is conversion of greenhouse gases into feedstocks within a circular economy, where resources recovered from waste become feedstocks for renewable energy and valuable products. Waste streams are also an information resource that can be recovered, deciphered, and used to inform public health decisions.

### (i) Managing landfill methane emissions.

As endpoints for municipal solid waste, landfills must be designed and vigilantly monitored to prevent the escape of methane. Monitoring and up-to-date models are needed to ensure that rates of methanogenesis do not exceed the rates of methane oxidation. Aboveground methanotrophic bioreactors can potentially convert recovered methane into valuable products, such as single-cell protein or bioplastics, while also generating methanotrophic biomass that can be incorporated into landfill cover soil. Challenges to the design and operation of such bioreactors are mass transfer limitations due to the low solubility of methane and oxygen, safe management of methane/oxygen mixtures, and provisions for heat management (24).

### (ii) Sequestering carbon via methane.

In urban environments, organic carbon is transported via sewers and trucks to wastewater treatment facilities and landfills, respectively. Humans and domestic animals produce vast quantities of fecal matter, projected to be 4.6 gigatons of dry waste globally/year by 2030, with animals producing six times that of humans (25). Other urban carbon streams include food waste (~0.5 gigatons of carbon [GtC]/year) and paper/cardboard wastes (0.2 GtC/year). Assuming that collection of these streams and their conversion to methane (waste organic composition: 50% carbon, 80% biodegradable, 90% converted to methane) yields 2.2 GtC as CH_4_ per year. The potential for carbon sequestration from such streams by pyrolytic conversion of methane to elemental carbon is thus on par with NOAA estimates of the global carbon land sink (2.6 GtC/year) (https://gml.noaa.gov/outreach/behind_the_scenes/gases.html). Recent advances in anaerobic secondary treatment of wastewater have demonstrated a net energy-positive operation in temperate climates (26). Central to this technology is the retention of acetoclastic methanogens attached to activated carbon particles and the use of ultrafiltration membranes to filter water and retain organic particles for hydrolysis and production of additional methane. Even more methane can be produced by feeding hydrogen and CO_2_ to hydrogenotrophic methanogens. The combination of high-rate methanogenesis and methane pyrolysis could enable recovery of carbon as graphene for a diverse range of applications in urban environments (27).

### (iii) Nitrous oxide mitigation.

Nitrous oxide is a potent greenhouse gas and the most significant ozone-depleting agent in the stratosphere. Under aerobic conditions, it is a by-product of ammonia oxidation mediated by ammonia-oxidizing archaea and bacteria. Under denitrifying (anoxic) conditions, N_2_O is produced by coupling NO reduction to oxidation of electron donors, such as Fe(II), sulfide, and sulfur, and organics. Research is needed to identify and quantify N_2_O production and consumption mechanisms within critical environments (estuaries, soils, landfills, and wastewater bioreactors). In conventional wastewater treatment plants designed for N removal, peak N_2_O emissions occur in low-oxygen transition regions. Strategies are needed to mitigate these emissions and to ensure reduction to N_2_. Possible solutions could include stripping of dissolved N_2_O into the gas phase and its use as a cooxidant with O_2_ of biogas methane. Another strategy is to provide electron donors sufficient for efficient denitrification and to ensure that species expressing N_2_O reductase are present. Potentially valuable metrics would include the relative ratios of gene expression for NO reduction to N_2_O (*qnor* + *cnorB*) (28) and for N_2_O reduction to N_2_ (*nos*Z) (29). To prevent N_2_O emissions, replacement of conventional aerobic treatment systems with energy-efficient anaerobic systems could enable the beneficial use of the effluent ammonia as fertilizer, offsetting demand for the Haber-Bosch process.

### (iv) Information from wastewater.

Climate change and its associated heat, flooding, diminished water quality, and disease vectors can bring increased disease transmission, particularly in densely populated environments. Monitoring of pathogens in domestic wastewater, as revealed by the COVID pandemic, is proving to be a valuable tool for monitoring disease transmission. Genetic tracking of SARS-CoV-2 and its variants correlates well with clinical data. Such monitoring can potentially enable early detection of bacterial or viral disease outbreaks, enabling better informed and more timely decision-making. Antibiotic resistance genes (ARGs) can also be measured. For pathogens and ARGs, climate change is thus a “threat multiplier” that drives dispersal of both pathogens and ARGs (30). Research is needed to determine how effective and extensive pathogen surveillance can be in managing disease outbreaks, including those driven by climate change.

## CLIMATE CHANGE AND MICROBES IN PUBLIC HEALTH

Microbes are much more adaptable and opportunistic than we humans. As microbes respond to climate change, their invisibility in our daily lives obscures their potential to increase the cost and burden of infectious and chronic diseases. Although the vast majority of bacteria, viruses, and fungi do not cause disease, climate change has led to geographic shifts of all organisms, resulting in unprecedented interactions among hosts, vectors, and microbes. Warmer temperatures, droughts, and weather extremes have led to the emergence of new pathogens, such as Candida auris, which may have become thermally adapted for growth in the human body (31). Other fungi previously thought to be nonpathogenic are now increasingly implicated in the incidence of fungal diseases that are antibiotic resistant and highly invasive (32).

Warmer temperatures affect the densities of airborne microbes and can accelerate their long-distance transport (33). Higher temperatures and environmental stresses can also alter human and animal physiologies and defenses against pathogens. Skin and gut microbiomes may become less protective. Exposures to zoonotic pathogens from wildlife, termed “spillover,” carry an additional risk of “spillback,” where the pathogen is reintroduced from humans to animals and undergoes mutations to pose new disease threats. Interactions between microbes and weakened hosts may induce bacteria to switch from “normal” to “persister” subpopulations as a “bet-hedging” strategy, resulting in antibiotic resistance or niche expansion (34). Diverse pathogenic microorganisms possess genetic elements that can be exchanged to increase infectivity and facilitate colonization of new niches. Increased monitoring and research on pathogen responses to climate change and their impacts on host-pathogen interactions will be crucial for mitigating climate change impacts on public health.

Greater public awareness of microbes’ opportunistic adaptability should lend more urgency to efforts to combat climate change. Better public understanding of the linkage between climate change and health threats could be assisted by the promotion of pathogen surveillance. Wastewater surveillance systems, such as those used to detect COVID-19, could continue to be enhanced. Real-time water or air monitoring programs for infectious agents could also be planned for implementation when climate models predict regional temperature shifts. A recent report by the IPCC now states with “high confidence” that climate-sensitive aquatic pathogens like *Vibrio* spp. have increased regional risks of water and foodborne disease. Data and documentation from surveillance efforts, which enabled the development of tools like the *Vibrio* Map Viewer in response to the northern expansion of *Vibrio* in Atlantic waters, will enable federal agencies to provide stronger guidance and early warnings of public health threats.

Publicity and outreach about the human microbiome could spur promotion of public awareness of microbial health threats from climate change (35). Education and research based on a more holistic, “One Health” way of thinking could help people acknowledge microbial threats and apply this knowledge to public health surveillance and protection. Explicit inclusion of “microbes” in definitions of “One Health,” for example, would affirm the need to recognize microbes as integral components of our environment and forces to be reckoned with. Currently, no common definitions of “One Health” include the word “microbes” (36). Perhaps they should, so that microbial awareness can be sustained to keep public health protection as fundamental motivation for combating climate change.

## MICROBES IN MODELS: BRIDGING THE GAP THROUGH INNOVATION

Metagenomics and other omics technologies hold wide potential to provide the necessary data inputs to inform climate models and pathogen surveillance efforts under global warming scenarios (37). The recovery of microbial genomes directly from a given environment through large-scale sequencing has provided a sweeping view into microbial diversity and functional potential (38, 39). However, this potential has yet to be translated into applications for climate science and addressing the pressing impacts of climate change. To facilitate this translation, bold and innovative actions are needed to expand the toolkit of high-throughput measures of microbial functions and metabolic rates *in situ*, to develop and advance mathematical modeling with considerations of microbial scale, and to fundamentally shift data infrastructure and data sharing practices to holistically support rapid dissemination, use, and knowledge extraction from microbiome data.

Metabolic dynamics and phenotypic properties of microbial communities are poorly understood. This knowledge gap has limited the incorporation of microbial parameters into climate models, despite microbes’ mediation of key steps in all biogeochemical cycles. An understanding of how microbes actively cycle nutrients, interact across species, and respond to disturbances (e.g., fires or extreme weather events) could offer insights into quantifying metabolically relevant features. New molecular assays to measure metabolic rates *in situ* and in high-throughput resolution could transform how we monitor microbiomes. Similarly, new experimental and statistical approaches to associate genomes from microbial isolates to community-level metabolic phenotypes hold the potential to leverage metagenome data to infer dynamic processes (40). Further, hypothesis-driven, mechanistic studies will support a predictive understanding of how microbes impact ecosystem processes beyond a descriptive framework (41).

The scale at which microbes operate presents another challenge for climate modeling. Microbes naturally function at the submicrometer scale yet collectively are estimated to contribute over 90 GtC (42). There is a pressing need to develop a theoretical framework and mathematical techniques to explicitly associate spatial, temporal, and phylogenetic factors with microbial scale and community assembly. Microbial community assembly is understood to be a mix of deterministic processes (e.g., selective pressures imposed by abiotic and species interactions) and stochastic processes (e.g., neutral dispersal, colonization, or extinction events), with different mechanisms dominating at different scales. Developing new mathematical techniques to delineate processes impacting assembly and scale will enable translation of microbial metabolic dynamics to ecosystem and global models. Furthermore, these new mathematical techniques can inform what microbiome measurements are necessary for long-term environmental monitoring, thereby forming a set of “microbial indicators” of climate change.

Paramount to advancing innovative new tools is the critical need for data infrastructure and support for open data sharing practices. Long-term research programs have made significant environmental monitoring investments, including the U.S. Department of Agriculture (USDA) Long-Term Agroecosystem Research (LTAR) Network, the National Science Foundation (NSF) LTER Program and NEON, the Department of Energy (DOE) Next-Generation Ecosystem Experiments (NGEE)-Arctic and Spruce and Peatland Responses Under Climatic and Environmental Change (SPRUCE), and the International Consortium of Ocean Observatories (Ocean Sites). All of these research programs include microbiome measurements and experimentation, yet the lack of coordination for standardized methods and data streams across these facilities presents unnecessary barriers to integrate across studies and ecosystems. An immediate path forward would be to create a framework for coordinated microbiome protocols and data sharing infrastructure, analogous to what was established as part of the international, multidisciplinary Tara Oceans project (43). By taking swift and immediate action to standardize microbiome data generation, the research community can more readily provide the necessary data inputs for climate models. A data-driven approach and robust shared data infrastructure will advance integration of microbes into climate models, providing improved climate projections and potentially new mitigation strategies that will invariably benefit society.

## CONCLUSION

There is overwhelming evidence that microbes contribute to climate change. Perhaps the clearest example of how microbial life contributes to atmospheric changes was the oxygenation of our atmosphere in the early epochs of Earth’s geologic history. Today, microbes continue to be the major players in ongoing atmospheric changes at all levels, including terrestrial, oceanic, and urban areas. From the warmth of cow rumen to the melting soils of permafrost regions, the symbiotic coral system in the oceans, and the carbon wastes of our cities, microbial metabolism is producing and absorbing gases that can affect climate. Hence, microbial contributions to the carbon flows to and from the atmosphere must be considered in all models of climate change. The microbial world could become a critical ally in the efforts to ameliorate the consequences of human emissions of GHG, since it should be possible to promote changes in microbial activities in some or maybe many environments to consume more and produce less gases that contribute to the warming of the atmosphere.

The trio of relationships between microbes, climate change, and human well-being is in need of more research and collaboration across the disciplines to address complex issues. As microbes adapt to a warming world, they can have direct effects on human well-being through altered patterns of host-microbe interactions, changed microbial biogeography, and altered terrestrial, aquatic, and urban microbiology. It is important that we move beyond the descriptive and correlational studies of microbiomes. Instead, the field needs more statistically defensible, hypothesis-driven, mechanistic studies to advance our understanding of the roles of microbes in climate change and their responses to environmental drivers, whether they be natural or by human intervention. Because the GHG have different residence times in the atmosphere, heat trapping capacities, and amenability to (microbial) interventions, perhaps this should be considered in setting research priorities (44). The research community needs innovative tools, resourceful research networks and infrastructures, integrated climate models, and interoperable data and framework to advance our knowledge (45). Moreover, efforts to inform policies and educate the public need to take place concurrently to increase awareness and gather support (45). As an example, the American Academy of Microbiology is leading the effort in building a 5-year scientific portfolio to focus on these important aforementioned issues. Consequently, microbiologists must redouble efforts to tackle multiple fronts to ensure that all discussions about climate change include the contributions of microbial life to these processes.

## References

[1] IPCC. Climate change 2022: impacts, adaptation, and vulnerability. Contribution of Working Group II to the Sixth Assessment Report of the Intergovernmental Panel on Climate Change, in press. Cambridge University Press, Cambridge, United Kingdom.

[2] Crowther TW, van den Hoogen J, Wan J, Mayes MA, Keiser AD, Mo L, Averill C, Maynard DS. 2019. The global soil community and its influence on biogeochemistry. Science 365:772. doi:10.1126/science.aav0550. 31439761

[3] Guo X, Feng J, Shi Z, Zhou X, Yuan M, Tao X, Hale L, Yuan T, Wang J, Qin Y, Zhou A, Fu Y, Wu L, He Z, Van Nostrand JD, Ning D, Liu X, Luo Y, Tiedje JM, Yang Y, Zhou J. 2018. Climate warming leads to divergent succession of grassland microbial communities. Nat Clim Chang 8:813–818. doi:10.1038/s41558-018-0254-2.

[4] Guo X, Zhou X, Hale L, Yuan M, Ning D, Feng J, Shi Z, Li Z, Feng B, Gao Q, Wu L, Shi W, Zhou A, Fu Y, Wu L, He Z, Van Nostrand JD, Qiu G, Liu X, Luo Y, Tiedje JM, Yang Y, Zhou J. 2019. Climate warming accelerates temporal scaling of grassland soil microbial biodiversity. Nat Ecol Evol 3:612–619. doi:10.1038/s41559-019-0848-8. 30911147

[5] Yuan MM, Guo X, Wu L, Zhang Y, Xiao N, Ning D, Shi Z, Zhou X, Wu L, Yang Y, Tiedje JM, Zhou J. 2021. Climate warming enhances microbial network complexity and stability. Nat Clim Chang 11:343–348. doi:10.1038/s41558-021-00989-9.

[6] Guo X, Gao Q, Yuan M, Wang G, Zhou X, Feng J, Shi Z, Hale L, Wu L, Zhou A, Tian R, Liu F, Wu B, Chen L, Jung CG, Niu S, Li D, Xu X, Jiang L, Escalas A, Wu L, He Z, Van Nostrand JD, Ning D, Liu X, Yang Y, Schuur EAG, Konstantinidis KT, Cole JR, Penton CR, Luo Y, Tiedje JM, Zhou J. 2020. Gene-informed decomposition model predicts lower soil carbon loss due to persistent microbial adaptation to warming. Nat Commun 11:4897. doi:10.1038/s41467-020-18706-z. 32994415 PMC7524716

[7] Luo Y, Wan S, Hui D, Wallace LL. 2001. Acclimatization of soil respiration to warming in a tall grass prairie. Nature 413:622–625. doi:10.1038/35098065. 11675783

[8] Zhou J, Xue K, Xie J, Deng Y, Wu L, Cheng X, Fei S, Deng S, He Z, Van Nostrand JD, Luo Y. 2012. Microbial mediation of carbon-cycle feedbacks to climate warming. Nat Clim Chang 2:106–110. doi:10.1038/nclimate1331.

[9] Crowther TW, Todd-Brown KEO, Rowe CW, Wieder WR, Carey JC, Machmuller MB, Snoek BL, Fang S, Zhou G, Allison SD, Blair JM, Bridgham SD, Burton AJ, Carrillo Y, Reich PB, Clark JS, Classen AT, Dijkstra FA, Elberling B, Emmett BA, Estiarte M, Frey SD, Guo J, Harte J, Jiang L, Johnson BR, Kröel-Dulay G, Larsen KS, Laudon H, Lavallee JM, Luo Y, Lupascu M, Ma LN, Marhan S, Michelsen A, Mohan J, Niu S, Pendall E, Peñuelas J, Pfeifer-Meister L, Poll C, Reinsch S, Reynolds LL, Schmidt IK, Sistla S, Sokol NW, Templer PH, Treseder KK, Welker JM, Bradford MA. 2016. Quantifying global soil carbon losses in response to warming. Nature 540:104–108. doi:10.1038/nature20150. 27905442

[10] Carey JC, Tang J, Templer PH, Kroeger KD, Crowther TW, Burton AJ, Dukes JS, Emmett B, Frey SD, Heskel MA, Jiang L, Machmuller MB, Mohan J, Panetta AM, Reich PB, Reinsch S, Wang X, Allison SD, Bamminger C, Bridgham S, Collins SL, Dato G, Eddy WC, Enquist BJ, Estiarte M, Harte J, Henderson A, Johnson BR, Larsen KS, Luo Y, Marhan S, Melillo JM, Peñuelas J, Pfeifer-Meister L, Poll P, Rastetter E, Reinmann AB, Reynolds LL, Schmidt IK, Shaver GR, Strong AL, Suseela V, Tietema A. 2016. Temperature response of soil respiration largely unaltered with experimental warming. Proc Natl Acad Sci USA 113:13797–13802. doi:10.1073/pnas.1605365113. 27849609 PMC5137763

[11] National Academies of Sciences, Engineering, and Medicine. 2021. Exploring a dynamic soil information system: proceedings of a workshop. The National Academies Press, Washington, DC. 10.17226/26170.

[12] Borer ET, Harpole WS, Adler PB, Lind EM, Orrock JL, Seabloom EW, Smith MD. 2014. Finding generality in ecology: a model for globally distributed experiments. Methods Ecol Evol 5:65–73. doi:10.1111/2041-210X.12125.

[13] Gao Q, Wang J, Xue K, Yang Y, Xie J, Yu H, Bai S, Liu F, He Z, Ning D, Hobbie S, Reich PB, Zhou J. 2020. Stimulation of soil respiration by elevated CO_2_ is enhanced under nitrogen limitation in a decade-long grassland study. Proc Natl Acad Sci USA 117:33317–33324. doi:10.1073/pnas.2002780117. 33318221 PMC7777058

[14] Longhurst A. 2006. Ecological geography of the sea, 2nd ed. Academic Press, New York, NY.

[15] Hagstrom GI, Levin SA. 2017. Marine ecosystems as complex adaptive systems: emergent patterns, critical transitions, and public goods. Ecosystems 20:458–476. doi:10.1007/s10021-017-0114-3.

[16] Gruber N. 2011. Warming up, turning sour, losing breath: ocean biogeochemistry under global change. Philos Trans A Math Phys Eng Sci 369:1980–1996. doi:10.1098/rsta.2011.0003. 21502171

[17] Oliver ECJ, Donat MG, Burrows MT, Moore PJ, Smale DA, Alexander LV, Benthuysen JA, Feng M, Sen Gupta A, Hobday AJ, Holbrook NJ, Perkins-Kirkpatrick SE, Scannell HA, Straub SC, Wernberg T. 2018. Longer and more frequent marine heatwaves over the past century. Nat Commun 9:1324. doi:10.1038/s41467-018-03732-9. 29636482 PMC5893591

[18] Frölicher TL, Fischer EM, Gruber N. 2018. Marine heatwaves under global warming. Nature 560:360–364. doi:10.1038/s41586-018-0383-9. 30111788

[19] Solomon S, Plattner G-K, Knutti R, Friedlingstein P. 2009. Irreversible climate change due to carbon dioxide emissions. Proc Natl Acad Sci USA 106:1704–1709. doi:10.1073/pnas.0812721106. 19179281 PMC2632717

[20] Karl DM, Bates NR, Emerson S, Harrison PJ, Jeandel C, Llinas O, Liu K-K, Marty J-C, Michaels AF, Miquel JC, Neuer S, Nojiri Y, Wong CS. 2003. Temporal studies of biogeochemical processes determined from ocean time-series observations during the JGOFS era. *In* Fasham MJR (ed), Ocean biogeochemistry: the role of the ocean carbon cycle in global change, p 239–267. Springer, New York, NY.

[21] Boyd PW, Collins S, Dupont S, Fabricius K, Gattuso J-P, Havenhand J, Hutchins DA, Riebesell U, Rintoul MS, Vichi M, Biswas H, Ciotti A, Gao K, Gehlen M, Hurd CL, Kurihara H, McGraw CM, Navarro JM, Nilsson GE, Passow U, Pörtner H-O. 2018. Experimental strategies to assess the biological ramifications of multiple drivers of global ocean change—a review. Glob Chang Biol 24:2239–2261. doi:10.1111/gcb.14102. 29476630

[22] Hobbs RJ, Hallett LM, Ehrlich PR, Mooney HA. 2011. Intervention ecology: applying ecological science in the twenty-first century. Bioscience 61:442–450. doi:10.1525/bio.2011.61.6.6.

[23] National Academies of Sciences, Engineering, and Medicine. 2021. A research strategy for ocean-based carbon dioxide removal and sequestration. The National Academies Press, Washington, DC. 10.17226/26278. 35533244

[24] El Abbadi SH, Criddle CS. 2019. Engineering the dark food chain. Environ Sci Technol 53:2273–2287. doi:10.1021/acs.est.8b04038. 30640466

[25] Berendes DM, Yang PJ, Lai A, Hu D, Brown J. 2018. Estimation of global recoverable human and animal faecal biomass. Nat Sustain 1:679–685. doi:10.1038/s41893-018-0167-0. PMC10922008 38464867

[26] Shin C, Tilmans SH, Chen F, McCarty PL, Criddle CS. 2021. Temperate-climate energy-positive anaerobic secondary treatment of domestic wastewater achieved at pilot-scale. Water Res 204:117598. doi:10.1016/j.watres.2021.117598. 34478994

[27] Jana P, de la Peña O'Shea VA, Coronado JM, Serrano DP. 2011. Co-production of graphene sheets and hydrogen by decomposition of methane using cobalt based catalysts. Energy Environ Sci 4:778–783. doi:10.1039/c0ee00490a.

[28] Woo SG, Sewell HL, Criddle CS. 2022. Phylogenetic diversity of NO reductases, new tools for *nor* monitoring, and insights into N_2_O production in natural and engineered environments. Front Environ Sci Eng 16:127. doi:10.1007/s11783-022-1562-3.

[29] Shan J, Sanford RA, Chee-Sanford J, Ooi SK, Löffler FE, Konstantinidis KT, Yang WH. 2021. Beyond denitrification: the role of microbial diversity in controlling nitrous oxide reduction and soil nitrous oxide emissions. Glob Chang Biol 27:2669–2683. doi:10.1111/gcb.15545. 33547715

[30] Fouladkhah AC, Thompson B, Camp JS. 2020. The threat of antibiotic resistance in changing climate. Microorganisms 8:748. doi:10.3390/microorganisms8050748. PMC7285162 32429413

[31] Robert V, Cardinali G, Casadevall A. 2015. Distribution and impact of yeast thermal tolerance permissive for mammalian infection. BMC Biol 13:18. doi:10.1186/s12915-015-0127-3. 25762372 PMC4381509

[32] Nnadi NE, Carter DA. 2021. Climate change and the emergence of fungal pathogens. PLoS Pathog 17:e1009503. doi:10.1371/journal.ppat.1009503. 33914854 PMC8084208

[33] Drautz-Moses DI, Luhung I, Gusareva ES, Kee C, Gaultier NE, Premkrishnan BNV, Lee CF, Leong ST, Park C, Yap ZH, Heinle CE, Lau KJX, Purbojati RW, Lim SBY, Lim YH, Kutmutia SK, Aung NW, Oliveira EL, Ng SG, Dacanay J, Ang PN, Spence SD, Phung WJ, Wong A, Kennedy RJ, Kalsi N, Sasi SP, Chandrasekaran L, Uchida A, Junqueira ACM, Kim HL, Hankers R, Feuerle T, Corsmeier U, Schuster SC. 2022. Vertical stratification of the air microbiome in the lower troposphere. Proc Natl Acad Sci USA 119:e2117293119. doi:10.1073/pnas.2117293119. 35131944 PMC8851546

[34] Morawska LP, Hernandez-Valdes JA, Kuipers OP. 2021. Diversity of bet-hedging strategies in microbial communities—recent cases and insights. WIRES Mech Dis 14:e1544. doi:10.1002/wsbm.1544. 35266649 PMC9286555

[35] Cavicchioli R, Ripple WJ, Timmis KN, Azam F, Bakken LR, Baylis M, Behrenfeld MJ, Boetius A, Boyd PW, Classen AT, Crowther TW, Danovaro R, Foreman CM, Huisman J, Hutchins DA, Jansson JK, Karl DM, Koskella B, Mark Welch DB, Martiny JBH, Moran MA, Orphan VJ, Reay DS, Remais JV, Rich VI, Singh BK, Stein LY, Stewart FJ, Sullivan MB, van Oppen MJH, Weaver SC, Webb EA, Webster NS. 2019. Scientists' warning to humanity: microorganisms and climate change. Nat Rev Microbiol 17:569–586. doi:10.1038/s41579-019-0222-5. 31213707 PMC7136171

[36] Mackenzie JS, Jeggo M. 2019. The One Health approach: why is it so important? Trop Med Infect Dis 4:88. doi:10.3390/tropicalmed4020088. PMC6630404 31159338

[37] American Academy of Microbiology. 2011. Incorporating microbial processes into climate models: this report is based on a colloquium convened by the American Academy of Microbiology, February 21–23, 2011, in Dallas, TX. American Society for Microbiology, Washington, DC. https://www.ncbi.nlm.nih.gov/books/NBK561255/. 32865933

[38] Almeida A, Nayfach S, Boland M, Strozzi F, Beracochea M, Shi ZJ, Pollard KS, Sakharova E, Parks DH, Hugenholtz P, Segata N, Kyrpides NC, Finn RD. 2021. A unified catalog of 204,938 reference genomes from the human gut microbiome. Nat Biotechnol 39:105–114. doi:10.1038/s41587-020-0603-3. 32690973 PMC7801254

[39] Nayfach S, Roux S, Seshadri R, Udwary D, Varghese N, Schulz F, Wu D, Paez-Espino D, Chen IM, Huntemann M, Palaniappan K, Ladau J, Mukherjee S, Reddy TBK, Nielsen T, Kirton E, Faria JP, Edirisinghe JN, Henry CS, Jungbluth SP, Chivian D, Dehal P, Wood-Charlson EM, Arkin AP, Tringe SG, Visel A, Abreu H, Acinas SG, Allen E, Allen MA, Alteio LV, Andersen G, Anesio AM, Attwood G, Avila-Magaña V, Badis Y, Bailey J, Baker B, Baldrian P, Barton HA, Beck DAC, Becraft ED, Beller HR, Beman JM, Bernier-Latmani R, Berry TD, Bertagnolli A, Bertilsson S, Bhatnagar JM, Bird JT, IMG/M Data Consortium. 2021. A genomic catalog of Earth’s microbiomes. Nat Biotechnol 39:499–509. doi:10.1038/s41587-020-0718-6. 33169036 PMC8041624

[40] Gowda K, Ping D, Mani M, Kuehn S. 2022. Genomic structure predicts metabolite dynamics in microbial communities. Cell 185:530–546.e25. doi:10.1016/j.cell.2021.12.036. 35085485

[41] Prosser JI, Martiny JB. 2020. Conceptual challenges in microbial community ecology. Philos Trans R Soc Lond B Biol Sci 375:20190241. doi:10.1098/rstb.2019.0241. 32200750 PMC7133534

[42] Bar-On YM, Phillips R, Milo R. 2018. The biomass distribution on Earth. Proc Natl Acad Sci USA 115:6506–6511. doi:10.1073/pnas.1711842115. 29784790 PMC6016768

[43] Sunagawa S, Acinas SG, Bork P, Bowler C, Eveillard D, Gorsky G, Guidi L, Iudicone D, Karsenti E, Lombard F, Ogata H, Pesant S, Sullivan M, Wincker P, de Vargas C, Tara Ocean Coordinators. 2020. *Tara* Oceans: towards global ocean ecosystems biology. Nat Rev Microbiol 18:428–445. doi:10.1038/s41579-020-0364-5. 32398798

[44] Neubauer SC, Megonigal JP. 2015. Moving beyond global warming potentials to quantify the climatic role of ecosystems. Ecosystems 18:1000–1013. doi:10.1007/s10021-015-9879-4.

[45] American Academy of Microbiology. 2022. Microbes & Climate Change: Science, People, and Impacts: Report on an American Academy of Microbiology (Academy) Virtual Colloquium held on 5 November 2021: American Society for Microbiology, Washington, DC. https://www.ncbi.nlm.nih.gov/books/NBK560256/. doi:10.1128/AAMCol.Nov.2021. 35544665

